# The Peroxidation of Lipids, Cellular Senescence and Aging

**Published:** 2024-12-02

**Authors:** T Blake Monroe, Paul D Robbins, David A Bernlohr

**Affiliations:** Department of Biochemistry, Molecular Biology and Biophysics, Institute on the Biology of Aging and Metabolism, University of Minnesota-Twin Cities, Minneapolis, Minnesota, USA

**Keywords:** Lipid peroxides, Senescence, Aging, Oxidative stress

## Abstract

The inducers of cellular senescence as a determinant in organismal aging are complex and likely driven by a combination of hormonal and metabolic factors. Lipids have recently been implicated as inducers of cellular senescence *in vitro* and *in vivo* across human and animal models and more directly, the electrophilic products of lipid peroxidation have been shown in a number of systems to initiate the senescence program. This review summarizes recent research at the interface of lipid biology and senescence. The review will emphasize the types of electrophilic lipids that induce senescence and how lipid scavengers are used to alleviate senescence burden and combat age-related disease.

## INTRODUCTION

Senescence in whole organisms is characterized by a progressive loss of physiological function and integrity. Over time, cells suffer insults such as mitochondrial damage, double stranded DNA breaks, telomeric attrition and disruptions to proteostasis. Accrual of cellular damage over time can bring about several cell fates (e.g. apoptosis, necrosis, etc.), one of which is senescence. Cellular senescence is a cellular fate characterized by the confluence of processes including withdrawal from the cell cycle, cell enlargement, overexpression of cyclin-dependent kinase inhibitors (e.g., p16^ink4a^ and p21^Cip1^), loss of nuclear envelope protein Lamin B1 and secretion of an array of inflammatory factors referred to as the SASP. Cellular senescence is thought to play a prominent role in the etiology of a growing list of diseases associated with aging including cardiovascular disease, neurodegenerative diseases and cancer [1].

Cellular senescence is often triggered by one or more forms of cellular stress. Stressors can be endogenous factors such as telomeric attrition or exogenous factors such as genotoxic compounds or ionizing radiation [2]. Oxidative stress is such a physiological stressor long associated with both senescence and age-related pathologies [3]. In 1956, Denham Harmon outlined the “Free Radical Theory of Aging,” wherein he proposed that age-related degenerations can be “attributed basically to the deleterious side attacks of free radicals on cell constituents” [4]. Lipids that make up the phospholipid bilayer can react with free radicals to generate a diverse array of lipid electrophiles and such lipids have been broadly implicated as senescence inducers [5].

The connection between oxidative stress, cellular senescence and aging enjoys thorough coverage by literature reviews. This review uniquely examines biogenic electrophiles as senescence inducers, a less explored aspect distinct from oxidative stress, with implications for aging and therapeutic intervention. In this review, we will examine evidence relating to the association between lipid peroxidation and aging, electrophilic lipid peroxidation products as senescence inducers and scavenging of lipid peroxidation products as anti-aging therapies.

## LITERATURE REVIEW

### Lipid peroxidation potentiates cellular senescence and correlates inversely with longevity

**Figure 1:**
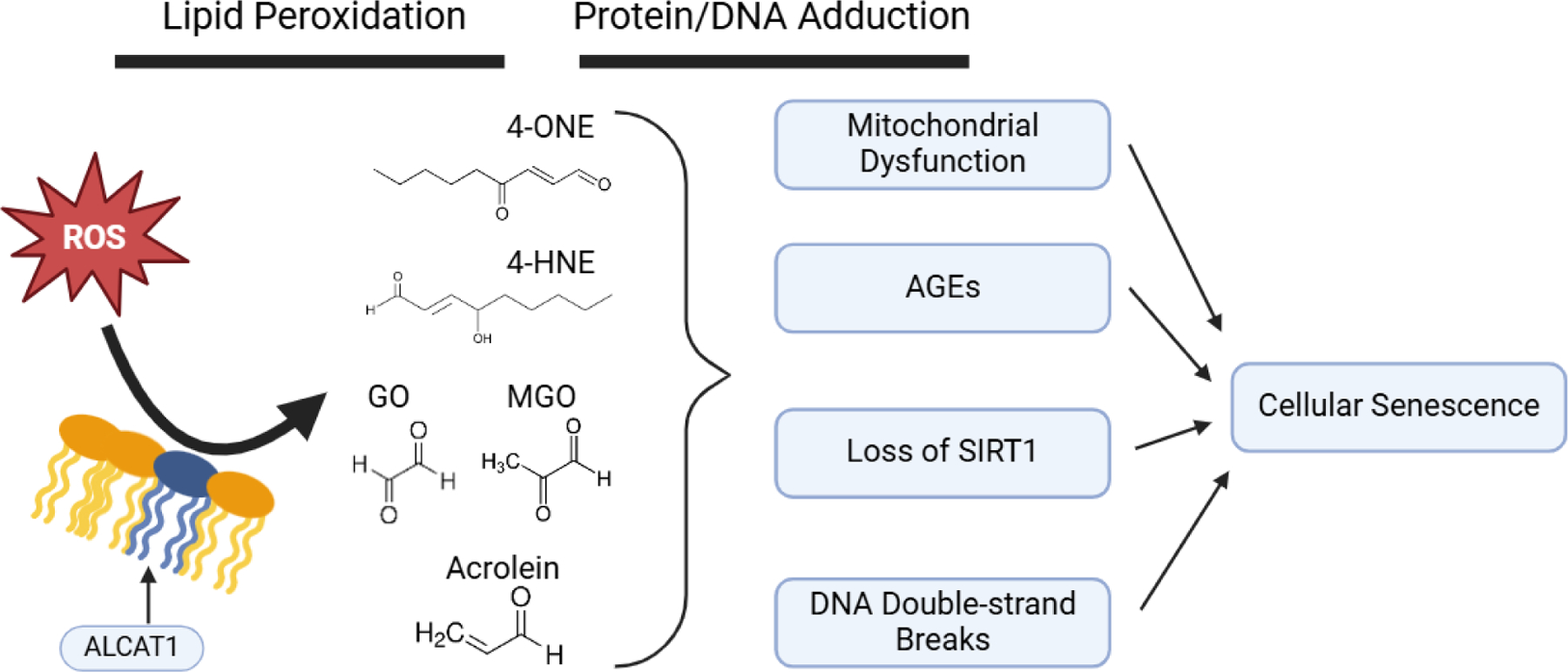
Graphical schematic of lipid electrophile biogenesis following peroxidation of the mitochondrial membrane and downstream effects leading to cellular senescence.

Lipid peroxidation occurs when radical oxygen species, produced in the course of normal metabolism, react with lipid species those that comprise the phospholipid membrane. Although this can physically occur anywhere in the cell, the high content of oxygen radicals in the mitochondrion makes this organelle a central node in lipid peroxidation of biological membranes [5]. Membrane lipids with acyl chains containing multiple double bonds (i.e., polyunsaturated fatty acids) are particularly susceptible to peroxidation due to the resonance stabilization of the resulting lipid radical. A major class of such compounds include α,β-unsaturated alkenals such as 4-HNE, 4-HHE, acrolein, crotonaldehyde and malondialdehyde. Another lipid class is dicarbonyls comprised of species such as 4-ONE, glyoxal and methylglyoxal (Figure 1). Due to the stochastic nature of radical reactions that mediate lipid peroxidation, the sum total of lipid-derived electrophiles are a heterogenous mixture of all these compounds [6].

Lipid peroxidation is recognized as a driver of cellular senescence, specifically for the manner in which peroxidation can remodel biological membranes [5]. As polyunsaturated fatty acids in the membrane are oxidized, the polarity they acquire cause them realign from the internal hydrophobic regions where they originate to the external aqueous side of the lipid bilayer. These protuberances have been termed “lipid whiskers” and they are posited to mediate misfolding of the mitochondrial membrane in senescent cells as well as immune cell recognition of senescent and apoptotic cells [7].

Lipid peroxidation and 4-HNE have been studied in the context of therapy-induced senescence [8]. Many topoisomerase inhibitors used in anti-cancer chemotherapies, including camptothecin and its derivatives, have been shown to induce lipid peroxidation and subsequent accumulation of excess lipid-derived electrophiles, including 4-HNE and 4-ONE. Through alkylation of a critical cysteine residue in the active site of topoisomerase I, 4-HNE can crosslink topoisomerase I and DNA in a covalent complex leading to double-stranded DNA breaks [9].

Recently, it’s been proposed that enhanced lipid peroxidation in various age-related disease is a consequence of adverse cardiolipin remodeling specifically by ALCAT1, an acyltransferase that produces cardiolipin with highly polyunsaturated conjugated acyl chains susceptible to lipid peroxidation [10]. In contrast, tafazzin carries out a similar acytransferase reaction but instead incorporates typically 18:2 acyl chains into cardiolipin that are more peroxidation resistant. Genetic loss of tafazzin (Barth Syndrome) leads to cardiolipin peroxidation and high amounts of 4-HNE [11]. A body of work implicates ALCAT1’s role in an array of pathologies associated with aging (Figure 1). In contrast, loss of function of ALCAT1, either *via* pharmacological or genetic means, results in amelioration of a number of age-related diseases including obesity, type 2 diabetes, heart failure and diabetic cardiomyopathy as well as a concomitant lowering of HNE adduction [10].

The susceptibility of biological membranes to lipid peroxidation is acknowledged as an important determinant of longevity [12,13]. Famously long-lived species such as the naked mole rat and the ocean quahog have been observed to possess mitochondrial membranes that are especially resistant to lipid peroxidation due to lower n-3 and n-6 polyunsaturated fatty acid content [13,14]. In comparing strains of mice, others have reported that lower n-3 PUFA content in the phospholipid profiles of muscle and liver resulted in longer lifespans, an effect that was attributed to reduced lipid peroxidation [12]. In humans, centenarians have repeatedly been found to possess lipidomes that are less susceptible to peroxidation than their elderly counterparts, underscoring the significance of lipid peroxidation in longevity [15].

### Lipid-derived electrophiles induce cellular senescence

Biogenic electrophiles have the ability to modify biomolecules with nucleophilic moieties such as nucleic acids and proteins (Figure 1). Lipid electrophiles will react with primary amines to form Schiff bases; this involves the addition of an aldehyde to an amine to make an imine. The predominant lipid elecrophile-protein products are Michael adducts wherein nucleophilic attack occurs on the beta carbon in the alpha/beta unsaturated chain. Further rearrangement can result in stable imidazole and hemi-acetal groups linking the peptide foundation to the akyl chain of the lipid electrophile [16]. Detection of reduced 4-HNE Michael adducts through the use of an antibody is frequently utilized to index biosynthesis of 4-HNE *via* Western blotting or ELISA [17]. Proteins that are alkylated by lipid electrophiles represent a major form of proteotoxicity and are targeted for degradation by the proteasomal apparatus [18]. Often, lipid alkylation will result in a loss in function for an adducted enzyme, as thiol and lysine groups in active sites tend to participate in chemistry critical for enzymatic catalysis. Accumulation of 4-HNE protein adducts have been observed in cellular senescence models *in vitro* as well as aging models *in vivo* in multiple tissue types [19,20].

Aside from amino acid side chains alkylation, 4-hydroxyalkenals can also form exocylic adducts with nucleic acids. 4-HNE can make Michael adducts directly with nucleosides to form a cyclized proprano-adduct. Alternatively, epoxidation of 4-HNE through prior autoxidation or interaction with other peroxides imparts the capacity to form etheno-adducts [21]. Since these etheno-adducts are removed through base excision repair and excreted from the cell, they are under investigation as potential biomarkers for DNA damage caused by 4-HNE and other biogenic aldehydes [22]. Multiplicity of reactive sites on some lipid electrophiles confers the ability to cross-link biomolecules (Figure 1). Aberrant protein aggregates, DNA crosslinks and protein-DNA complexes are all known features of several pathologies [23].

While oxygen radicals such as superoxide have biological half-lives measured in micro or milliseconds, these biogenic electrophiles can exist in their free state for minutes to hours. Therefore, these compounds can, theoretically, exert effects far more distant from their site of genesis than radical oxygen species. Excess 4-HNE adduction has been observed in multiple models of senescence, including replicative and irradiative senescence, posing the possibility that lipid-derived electrophile generation is a feature of multiple modes of senescence [19,24]. Direct exposure to lipid-derived electrophiles themselves cause cells to senesce *in vitro*, as documented in a body of work summarized as follows.

#### 4-Hydroxynonenal and 4-Oxo-2-Nonenal:

The chemistry of medium chain lipid-derived enals, particularly 4-HNE, has been well characterized and 4-HNE is known to exert genotoxicity and proteotoxicity through induction of carbonyl stress, a shared feature of multiple degenerative pathologies. 4-HNE and 4-ONE have been shown to induce cellular senescence in a number of cell culture models. A recently published study using IMR90 fibroblasts and murine adipose progenitor cells demonstrated the capacity of 4-HNE and 4-ONE to induce cellular senescence and provided detailed characterization of the accompanying senescence program, carbonyl stress, mitochondrial dysfunction and genotoxicity. The authors of this study termed this senescence program BLIS [25]. The observed incidence of BLIS spans multiple cells models and species, including human placental cells, human foreskin fibroblasts and bovine endothelial cells [26–28].

A comprehensive understanding of the molecular mechanism accounting for BLIS remains elusive due to the pleiotropic phenotype associated with BLIS and the stochastic nature of 4-HNE adduction.

#### Malondialdehyde:

MDA is a dicarbonyl that tautomerizes to an enol. MDA is widely used as a biomarker of lipid peroxidation. Post-translational modification of proteins by MDA is thought to play a role the etiology of many age-related disease states [29]. However, to our knowledge, MDA has not been demonstrated to directly induce senescence *in vitro* and thus, molecular mechanisms tying MDA to cellular senescence remain an area for active investigation.

#### Glyoxal and methylgloxal:

GO is a diketal byproduct of lipid peroxidation or the autoxidation of glucose. GO reacts with proteins to form AGEs, one of which is CML, a common biomarker used to index AGE biosynthesis. At concentrations ranging from hundreds of micromolar to millimolar, GO causes cellular senescence in human skin fibroblasts and bone-marrow derived, immortalized mesenchymal stem cells, as well as a concomitant increase in CML [30,31].

MGO, a related dicarbonyl produced in lipid peroxidation and glycolysis, induces senescence in murine adipose-derived stem cells. In cocultures with endothelial cells, stem cells made senescent with MGO were found to have hampered pro-angiogenic signaling capacity [32].

#### Acrolein:

Acrolein is the simplest unsaturated alkene and is may be generated endogenously as a byproduct of lipid peroxidation or introduced exogenously as an environmental toxicant commonly found in air pollution and cigarette smoke. Acrolein has been shown to induce cellular senescence and accelerated telomere shortening in two different lines of human lung fibroblasts [33,34].

### Scavenging biogenic electrophiles attenuates senescence

Abatement of carbonyl stress *via* direct sequestration or enhancement of biogenic electrophile disposal enzymes has been shown to effectively decrease senescence burden and ameliorate age-related disease.

#### TA293:

Cytosolic hydroxyl radicals were found to generate oxidized phospholipids, which in turn, were determined to enact cellular senescence independent of TLR4 signaling and inflammatory response activation. In a mouse model of enhanced oxidative stress, administration of a small molecule hydroxyl radical scavenger (TA293) normalized levels of oxidized phospholipids and blunted expression of senescence markers SA-β-galactosidase, p21 and p16 in lung and kidney [35].

#### Aminoguanidine:

Aminoguanidine is a scavenger of dicarbonyls such as GO and MGO. In two different strains of rat, aminoguanidine was demonstrated to attenuate age-related declines in renal and cardiovascular function, presumably through prevention of AGE accumulation. In primary human vascular endothelial cells, glyoxal-induced senescence as measured by p21 expression and formation of CML was abrogated with coadministration of aminoguanidine [36].

#### Glyoxalase 1:

GLO1 is a member of the glyoxalase enzyme system that detoxifies glyoxal by conjugation to glutathione. Aged rats that overexpressed GLO1 exhibited lower closed proteins in renal tissue compared to their wild-type counterparts, coincident with reduced senescence markers (p53, p21 and p16) as well as improved markers of kidney function. Experiments done *in vitro* in the same study demonstrated that overexpression of GLO1 attenuates markers of senescence induced through exposure to the genotoxic topoisomerase poison, etoposide [37]. These results suggest that excessive glyoxal generation could be a common feature of multiple models of cellular senescence.

#### Ethanol:

Exposure of human aortic endothelial cells to ethanol partially rescued serially passaged cells from replicative senescence through induction of ALDH2. As ALDH2 is a major detoxification enzyme for biogenic electrophiles, an increase in ALDH2 activity would be expected to relieve carbonyl stress. Indeed, it was found that ALDH2 induction through moderate ethanol exposure resulted in lower 4-HNE adducts. Of note, it was found that ethanol promoted nuclear translocation of SIRT1 [38].

#### Carnosine:

L-carnosine is a dipeptide of histidine and beta alanine that’s abundant in millimolar concentrations in tissues with high bioenergetic demand such as skeletal muscle and brain. It is an aldehyde scavenger that makes an irreversible adduct with 4-HNE and other biogenic aldehydes, effectively acting to sequester reactive electrophilic moieties. Tissue quantity of L-carnosine is regulated by carnosine synthase and carnosinase, which biosynthesize and degrade carnosine, respectively.

L-carnosine has been shown to have anti-senescence effects *in vitro* [39]. Concurrent treatment of fibroblasts undergoing BLIS with a stoichiometric excess of L-carnosine with continuous 4-HNE exposure results in amelioration of the senescent phenotype as measured by SA-β-galactosidase activity and p21 expression [25]. Culturing fibroblasts with L-carnosine reduces 8-hydroxyguanine adducts upon starvation challenge, protects serially passaged cells from oxidative stress and telomeric shortening and rescues UV-exposed cells from DNA double stranded breaks and SIRT1 loss [40–43].

Studies *in vivo* evaluating degenerative disease have also seen benefits from carnosine. In mice fed a high fat and high sugar diet, L-carnosine administered in drinking water both improved metabolic parameters and reduced markers of senescence in adipose [25]. In a progeroid mouse model (SAMP8), treatment with L-carnosine rescued mitochondrial function and attenuated senescence-associated cognitive decline [44]. A 2023 meta-analysis of nine clinical studies testing the efficacy of L-carnosine against age-related disease concluded that L-carnosine demonstrated clear benefits in ameliorating type 2 diabetes and neurodegenerative disorders [45].

## DISCUSSION

Capacity to metabolize lipid-derived electrophiles seems to play a significant role in age-related disease and longevity. A major constituent of the electrophile disposal apparatus is GSTs, a family of enzymes that metabolize biogenic aldehydes by conjugating them to thiols in glutathione. In C elegans, ectopic expression of murine glutathione-s-transferase A4 or overexpression of glutathione-s-transferase 10 were associated with a longer lifespan while gene silencing of glutathione-s-transferase 10 was associated with a shorter one; an effect was recapitulated in mice [46]. In a study of the transcriptomes associated with 15 lifespan-lengthening interventions in mice, the gene most frequently upregulated (in 9 interventions) was GstA4 [47]. To our knowledge, biogenic electrophile disposal function has never been studied with regards to human longevity.

A significant obstacle to research in biogenic electrophiles as endogenous senescence inducers is the complexity of the molecular mechanisms underlying electrophile-induced senescence. Indeed, multitude cell signaling mechanisms are proposed to play a role in establishing the senescent phenotype.

Several interesting connections between senescence, disturbances in redox signaling, mitochondrial dysfunction and disruption of sirtuin signaling have been observed coincident with BLIS, brought on by exposure to medium chain lipid electrophiles such as 4-HNE and 4-ONE [25–28]. Mitochondrial dysfunction, itself an effector of cellular senescence, is a well-documented sequela of 4-HNE exposure [11]. One study found that part of BLIS is mediated by upregulation of pro-oxidant thioredoxin-interacting protein downstream of Peroxisome Proliferator Activated Receptor γ activation [28]. 4-HNE is known to possess the capacity to alkylate nuclear acetylase sirtuin 1, an inhibitor of p53 signaling and senescence. Interestingly, development of BLIS coincides with loss of SIRT1 and concurrent promotion of overall protein ubiquitination and acetylation [26]. Mitochondrial dysfunction and proteasomal degradation of acrolein-modified Werner’s syndrome protein, a helicase involved in telomere maintenance and DNA repair, mediate acrolein’s senogenic effects of acrolein [33].

The primary challenge of studying protein adducts of biogenic electrophiles are their diversity; characterization of post-translational modifications by lipid electrophiles are required for their quantification *in vivo*. Moving forward, researchers in the aging field can leverage increasingly complicated metabolomic, proteomic and transcriptomic technologies to fully elucidate complex molecular mechanisms basis biogenic electrophile-induced senescence.

## CONCLUSION

Through generation of extremely reactive lipid electrophiles, lipid peroxidation can initiate a cascade of biomolecular and cell signaling modifications that ultimately lead to cellular senescence and potentiation of age-associated pathologies. Lipid electrophiles represent a diverse set of endogenously-generated senogenic compounds that accumulate with age. Scavenging lipid electrophiles represents a potential therapeutic method for alleviating age-related and degenerative disease. Further work should be done to glean a more complete understanding of molecular mechanisms underlying lipid electrophiles’ adverse influence on physiological aging.

## Abbreviations:

4-HHE: 4-Hydroxyhexenal
4-HNE: 4-Hydroxynonenal
4-ONE: 4-Oxo-2-Nonenal
AGE: Advanced Glycation Endproduct
ALCAT1: Acyl-CoA:Lysocardiolipin Acyltransferase-1
ALDH2: Aldehyde Dehydrogenase 2
BLIS: Biogenic Lipid Induced Senescence
CML: Carboxymethyl Lysine
ELISA: Enzyme-Linked Immunosorbent Assay
FOXO3: Forkhead Box O3
GLO1: Glyoxalase I
GO: Glyoxal
GST: Glutathione-S-Transferases
MGO: Methylglyoxal
PPARγ: Peroxisome Proliferator-Activated Receptor Gamma
PUFA: Polyunsaturated Fatty Acid
TLR4: Toll-Like Receptor 4
TXNIP: Thioredoxin-Interacting Protein
SAMP8: Senescence-Accelerated Mouse-Prone 8
SASP: Senescence Associated Secretory Phenotype
SIRT1: Sirtuin 1
